# Chronic Cerebral Hypoperfusion-Induced Disturbed Proteostasis of Mitochondria and MAM Is Reflected in the CSF of Rats by Proteomic Analysis

**DOI:** 10.1007/s12035-023-03215-z

**Published:** 2023-02-21

**Authors:** Vanda Tukacs, Dániel Mittli, Éva Hunyadi-Gulyás, Dávid Hlatky, Katalin F. Medzihradszky, Zsuzsanna Darula, Gabriella Nyitrai, András Czurkó, Gábor Juhász, József Kardos, Katalin A. Kékesi

**Affiliations:** 1 ELTE NAP Neuroimmunology Research Group, Department of Biochemistry, Institute of Biology, ELTE Eötvös Loránd University, Budapest, Hungary; 2 Laboratory of Proteomics, Institute of Biology, ELTE Eötvös Loránd University, Budapest, Hungary; 3 Laboratory of Proteomics Research, Biological Research Centre, Eötvös Loránd Research Network, Szeged, Hungary; 4 Preclinical Imaging Center, Pharmacology and Drug Safety Research, Gedeon Richter Plc., Budapest, Hungary; 5 Single Cell Omics Advanced Core Facility, Hungarian Centre of Excellence for Molecular Medicine, Szeged, Hungary; 6 InnoScience Ltd., Mátranovák, Hungary; 7 Department of Biochemistry, Institute of Biology, ELTE Eötvös Loránd University, Budapest, Hungary; 8 Department of Physiology and Neurobiology, Institute of Biology, ELTE Eötvös Loránd University, Budapest, Hungary

**Keywords:** Chronic cerebral hypoperfusion, BCCAO, Proteomics, Mitochondria, MAM, CSF

## Abstract

Declining cerebral blood flow leads to chronic cerebral hypoperfusion which can induce neurodegenerative disorders, such as vascular dementia. The reduced energy supply of the brain impairs mitochondrial functions that could trigger further damaging cellular processes. We carried out stepwise bilateral common carotid occlusions on rats and investigated long-term mitochondrial, mitochondria-associated membrane (MAM), and cerebrospinal fluid (CSF) proteome changes. Samples were studied by gel-based and mass spectrometry-based proteomic analyses. We found 19, 35, and 12 significantly altered proteins in the mitochondria, MAM, and CSF, respectively. Most of the changed proteins were involved in protein turnover and import in all three sample types. We confirmed decreased levels of proteins involved in protein folding and amino acid catabolism, such as P4hb and Hibadh in the mitochondria by western blot. We detected reduced levels of several components of protein synthesis and degradation in the CSF as well as in the subcellular fractions, implying that hypoperfusion-induced altered protein turnover of brain tissue can be detected in the CSF by proteomic analysis.

**Supplementary Information:**

The online version contains supplementary material available at 10.1007/s12035-023-03215-z.

## Introduction

Chronic cerebral hypoperfusion (CCH) is a pathological state that is characterized by declining cerebral blood flow (CBF). One of the potential risk factors for vascular dementia (VD) and sporadic Alzheimer’s disease (AD) is the decreased blood flow of the brain [1, 2]. Bilateral common carotid artery occlusion (BCCAO) induces reduced cerebral blood flow, which results in oxidative stress, inflammatory response, and disturbed lipid metabolism leading to cognitive impairment [3]. In the stepwise BCCAO model, rats have 1 week of regeneration time between the bilateral common carotid artery occlusions, leading to a gradual decrease in cerebral blood flow. After 8 weeks of occlusion, the CBF is almost completely recovered in the frontal cortex [4]; however, cognitive deficits and oxidative stress are apparent [5]. In this study, we performed stepwise BCCAO and investigated the long-term effects of the model.

Brain plasticity is the ability of the brain to modify its structure and function in response to the alterations of its environment. Many subcellular organelles can adapt to changing environments of neurons such as synapses and mitochondria. In our previous studies, we have investigated mitochondrial [6] and mitochondrial-associated membrane (MAM) proteome [7] alterations in Alzheimer’s disease model animals, revealing the relevance of these organelles in neurodegeneration.

Since the brain is one of the most energy-demanding organs and its ATP source relies mostly on oxidative phosphorylation, mitochondrial proteome changes can give insight into its molecular adaptation to CCH. The mitochondria are vital, dynamic, and plastic organelles that are essential for maintaining membrane ion gradients, neurotransmission, and synaptic plasticity, requiring a large amount of energy. Moreover, mitochondria participate in other neuronal processes such as calcium buffering and intracellular signaling. Dysfunctions of mitochondria were implicated in VD and its animal models [5, 8, 9] and several other neurodegenerative disorders [10–13]. In the BCCAO model, increased mitochondrial DNA deletion and structural damage were observed [14]. Despite the known role of mitochondria in CCH, our study is the first proteomic analysis of this organelle in the BCCAO model.

Furthermore, mitochondrial dynamics are disturbed in several neurodegenerative disorders [11, 15]. MAM also participates in the protein supply of mitochondria and regulates mitochondrial dynamics [16]. Besides mitochondrial dynamics, MAM also regulates essential cellular processes, such as fatty acid metabolism and calcium homeostasis [17]. Also, it has a main role in the processing of amyloid precursor protein which is dysregulated in AD [18]. In addition, MAM dysfunction is involved in other neurodegenerative diseases [17]; however, its role in CCH is poorly studied.

Cerebrospinal fluid (CSF) has a vital role in the clearance of brain interstitial fluid [19]. Due to its direct contact with the brain, its content can reflect biochemical changes in the brain. CSF has detectable protein content; therefore, proteomic alterations in the CSF might provide potential biomarkers that can indicate early, pre-symptomatic pathological alterations, and CSF sampling is feasible in human translational studies.

Additionally, decreased brain metabolism is one of the earliest clinical symptoms of AD which induces mitochondrial dysfunction in the brain [20]. Detecting molecular shifts of mitochondria or MAM in the CSF can provide early and detectable pathological signs of neurodegeneration. In the current study, we aimed to monitor changes induced by BCCAO in the MAM and mitochondria of the frontal cortex which is one of the brain areas responsible for higher-order cognitive functions and affected in VD [21, 22]. We also analyzed the proteome of the CSF to reveal whether organellar changes could be detected in body fluids. We applied mass spectrometry-based (MS-based) and gel-based proteomic methods to study the proteomic alterations - induced by stepwise BCCAO - of CSF and the organelles of the frontal cortex, respectively.

## Materials and Methods

### Animals

Adult male Wistar rats (aged 3 months, weighing ~ 220 g) were obtained from Toxi-Coop Ltd. (Budapest, Hungary). Rats were housed under standard laboratory conditions (lights on at 9:00 AM, lights off at 9:00 PM) in temperature- and humidity-controlled rooms with ad libitum access to food and water. All animal care and experimental procedures were following the Council Directive 86/609/EEC, the Hungarian Act of Animal Care and Experimentation (1998, XXVIII). All the procedures conformed to the National Institutes of Health guidelines were in accordance with the guidelines of the local Animal Care and Use Committee and were approved by the local Ethical Committee of Gedeon Richter Plc. (PE/EA/2885–6/2016). All efforts were carried out to minimize the animals’ pain and suffering and to reduce the number of animals used. A total of 16 rats were used in the experiments; they were assigned randomly to operated and sham-operated groups. We guaranteed the blindness of the experimenters whenever it was possible.

### Stepwise Bilateral Occlusion of Common Carotid Arteries

Stepwise bilateral common carotid artery occlusion was performed on rats as previously described [7, 23, 24]. Briefly, rats were anesthetized with isoflurane (1.5–2% in air), and a ventral midline incision was placed on the neck. First, the left common carotid artery was exposed, gently separated from the vagus nerve, and occluded by three ligatures (2–0). The animals were allowed to regenerate for a week in their home cages. Then, the same surgical procedure was performed on the right common carotid artery too. Rats in the control group underwent a sham operation as they received the same surgical procedures in both steps, but only a thread was placed around the vessels without ligation of the arteries.

### Magnetic Resonance Angiography

The efficacy of the occlusions and the changes in cerebral blood flow were monitored with MRA, on the second and fifth weeks after the second occlusion, based on our previous studies [23, 24]. Anesthetized animals were scanned in a 9.4 T MRI system (Varian, Medical Systems Inc., Palo Alto, CA, USA) with a free bore of 210 mm, containing a 120 mm inner size gradient coil. Three-dimensional time-of-flight angiography (3D gradient echo) was performed at TR/TE = 30/2.8 ms, resolution = 0.42 × 0.42 × 0.46 mm. The MRA studies were performed to evaluate cervical and intracranial arteries and parenchymal injury. For proteomic experiments, the animals had to meet the criteria as follows: (i) lack of gross anatomical abnormalities (i.e., pathologically large or asymmetrical anatomical structures) and (ii) lack of extensive lesions or any sign of extended tissue impairment. The MRA revealed ischemic lesions in none of the animals. Maximum intensity projection was applied on the volumetric MRA recordings to create 2D images. The images were analyzed by ImageJ2; to discard background noise, the low threshold of 110 was applied on the intensity values. Then, the images were inverted, and mean gray values were obtained. A nonparametric, Mann–Whitney test was applied to the mean gray values of the MRA images with a significance level of 0.05.

### Collection of CSF and Preparation of Mitochondria and MAM Samples

We collected CSF and prepared mitochondria and the subcellular fraction containing MAM from the frontal cortices of sham-operated and operated rats based on the protocol of Wieckowski et al. [25]. Animals were anesthetized with isoflurane (1.5–2% in air), and CSF was collected from cisterna magna, then rats were sacrificed and their brains were quickly removed from the skull. Brain samples were washed in ice-cold isolation buffer-1 (IB-1) (225 mM mannitol, 75 mM saccharose, 0.5% BSA, 0.5 mM EGTA, and 30 mM Tris–HCl pH 7.4), then three times in ice-cold isolation buffer-3 (IB-3) (225 mM mannitol, 75 mM saccharose, and 30 mM Tris–HCl pH 7.4). Brain samples were cut into smaller pieces and washed in IB-1. Brain samples were homogenized in IB-1 supplemented with protease and phosphatase inhibitor cocktail with a Dounce tissue grinder (Sigma-Aldrich) manually (15 strokes per sample). Homogenized samples were centrifuged at 740 × g for 7 min at 4 °C. Supernatants were centrifuged at 9000 × g for 10 min at 4 °C, and pellets were suspended in isolation buffer-2 (IB-2) (225 mM mannitol, 75 mM saccharose, 0.5% BSA, and 30 mM Tris–HCl pH 7.4). Samples were centrifuged at 10,000 × g for 10 min at 4 °C, and pellets were suspended in IB-3. Samples were centrifuged again at 10,000 × g for 10 min at 4 °C, and pellets were suspended in mitochondria resuspending buffer (MRB) (250 mM mannitol, 5 mM HEPES (pH 7.4), and 0.5 mM EGTA); this is the crude mitochondrial fraction that contains MAM. One-fourth of each sample was centrifuged at 10,000 × g for 10 min at 4 °C, and pellets were resuspended in lysis buffer (7 M urea, 2 M thiourea, 4% CHAPS, 20 mM Tris, 5 mM magnesium-acetate). The other three-fourths of the fractions were used for pure mitochondria preparation. Samples were layered onto Percoll medium and centrifuged at 95,000 × g for 35 min at 4 °C with SW-40 Ti rotor. The pure mitochondria fraction of the sample was collected from the interface and centrifuged at 6300 × g for 10 min at 4 °C. Pellets were resuspended and centrifuged again at 6300 × g for 10 min at 4 °C with 70.1 Ti rotor, and pellets were resuspended in lysis buffer. All the samples were stored at − 80 °C until use.

### Proteomic Investigation of Subcellular Fractions

We investigated the proteome alterations of MAM-containing and mitochondria samples from the frontal cortex of 6 sham-operated and 6 BCCAO rats, using two-dimensional differential gel electrophoresis (2-D DIGE). The samples were adjusted to pH 8 and pH 8.5, respectively. Then, their protein concentration was measured by the 2D-Quant kit (GE Healthcare, Chicago, IL, USA). The fluorescent labeling of the mitochondrial proteins was conducted with a CyDye DIGE Fluor Minimal dye labeling kit (Cytiva). From BCCAO and control rats, 50 µg of protein of each sample were randomly labeled with either Cy5 or Cy3 dyes, while the internal sample (containing equal protein amounts (25 μg)) was labeled with Cy2. MAM-containing samples were labeled with CyDye DIGE Fluor labeling kit for Scarce sample (Cytiva). Five µg of protein from each sample and the internal standard were labeled with Cy3 and Cy5, respectively. The differently labeled samples were mixed and rehydrated passively onto Immobiline DryStrip gel strips (24 cm, pH 3–10 NL, GE Healthcare) overnight. Isoelectric focusing (IEF) was performed in an EttanIPGphor 3 IEF unit for 24 h to attain a total of 100 kVh (GE Healthcare). Following IEF, the mitochondrial proteins were reduced and carbamidomethylated using an equilibration buffer containing 1% mercaptoethanol and 2.5% iodoacetamide, respectively. In the case of MAM, proteins were only reduced as previously mentioned. SDS-PAGE separation was performed on 24 × 20 cm, 10% polyacrylamide gels in an EttanDALTsix Electrophoresis System (GE Healthcare). Then, the gels were scanned with a TyphoonTRIO + scanner (GE Healthcare) using appropriate lasers and filters with the photomultiplier tube biased at 600 V. Differential protein analysis was performed using the DeCyder v7.0 software package (GE Healthcare), employing its differential analysis and biological variance analysis modules. The fluorescence intensities of the Cy3 and Cy5 dyes on a particular gel were normalized to the intensity of the Cy2 dye, or Cy3 intensities were normalized to the Cy5 dye. Quantitation of the fluorescence intensities of the protein spots and statistical analyses were carried out using the software. Independent two-tailed Student’s *t*-test was performed on spots that are present on at least 80% of the gels and have fold changes of more than ± 1.2. The Benjamini–Hochberg procedure was applied with a false discovery rate of 0.25. In the results, we show the original *P*-values of only those spots that remained significant after the procedure. Statistically significantly altered protein spots (*P* < 0.05) were picked for further protein identification. For the identification of proteins in spots of interest, preparative 2-D gel electrophoresis was performed separately using a total of 800 μg of synaptic proteins per gel. Resolved protein spots were visualized by the Colloidal Coomassie Blue G-250 stain (Merck Millipore, Billerica, MA, USA). One preparative gel for each brain region was run, and the selected spots were manually excised from the gels with pipette tips for protein identification. Excised spots were placed in a 1% acetic acid solution.

### Mass Spectrometry-Based Protein Identification from 2-D Gel Spots

Proteins in the selected 2-D gel spots were in-gel digested as described in the protocol available online (http://msf.ucsf.edu/proto cols.html). Briefly, gel spots were cut into smaller cubes, washed with 25 mM ammonium-bicarbonate/50% acetonitrile, reduced using 10 mM TCEP and alkylated with 55 mM MMTS. After dehydration, the gel pieces were rehydrated with 100 ng, trypsin )sequencing grade, side chain protected porcine trypsin, Promega) in 20 μl of 25 mM ammonium-bicarbonate. Samples were digested for 4 h at 37 °C. Tryptic peptides were extracted and dried in a vacuum centrifuge.

Samples were reconstructed in 20 µl of 0.1% formic acid before mass spectrometric analysis. Five µl of the digest was injected for LC–MS/MS analyses onto an LTQ-Orbitrap Elite (Thermo Fisher Scientific) mass spectrometer online coupled with a nanoAcquity UPLC (waters) system. In order to shorten the injection time, the sample was injected with a high flow rate to a trap column (Symmetry C18, nanoACQUITY UPLC 2D, V/M 0.180 mm × 20 mm, 5 µm, 100 Å, waters,) and a nano column (BEH130, C18 Acquityuplc column, 0.100 mm × 100 mm, 1.7 µm,130 Å, waters) was used for the analysis. Gradient elution was applied from 3 to 40% of eluent B (0.1% formic acid in acetonitrile) in 37 min. Mass spectrometry data were collected in a data-dependent manner; a high-resolution survey scan was followed by a maximum of 20 dependent CID spectra analyzed in the ion-trap. Only multiple charged precursor ions were selected for fragmentation, and after that, they were excluded for 30 s from the repeated selection. A PAVA script (UCSF, MSF, San Francisco, CA) was used for peak picking and our *in-cloud*ProteinProspector (version: 5.22.0) server (https://cloud.mta.hu/) was used for database search, with the following parameters: UniProtKB.2019.6.12.random.concat database was filtered for the rat sequences concatenated with the most frequent contaminant proteins (36,319 sequences; with 209 additional contaminant protein sequences were searched). Only tryptic peptides were considered, with a maximum of one missed cleavage site. Several variable modifications were set as acetyl (protein N-term), acetyl + oxidation (protein N-term M), Gln- > pyro-Glu (N-term Q), Met-loss (protein N-term M), Met-loss + acetyl (protein N-term M), oxidation (M), and carbamidomethyl- and methyltio-cysteine. Mass accuracy was set to 10 ppm for the parent and 0.6 Da for fragment ions. Proteins and peptides were accepted with a maximum of 1% FDR. Proteins were rejected with less than 10 unique peptides, 30 peptide counts, or 40% of sequence coverage; accepted proteins are shown in Table 1.

### CSF Proteome Analysis Using LC–MS/MS

The protein content of the collected rat cerebrospinal fluid samples was treated with trypsin using the S-Trap micro spin columns according to the vendor’s protocol (https://protifi.com/pages/protocols), and the resulting peptide mixture was analyzed using a Waters MClass nUPLC-Thermo Orbitrap Fusion Lumos Tribrid LC–MS system in a data-dependent fashion. Proteins were identified using the Protein Prospector BatchTag Web software applying score-based acceptance criteria. In more detail, the tryptic digest was injected onto a trapping column (MClass Symmetry Waters Acquity UPLC Trap Col 2G V/M C18 column, 0.180 mm ID * 20 mm L, 5 m particle size, 100 Å pore size; loading time: 3 min with 1% B at 5 l/min) and after desalting was separated using a nonlinear gradient of 10–50% B in 80 min (solvent A: 0.1% formic acid/water, solvent B: 0.1% formic acid/acetonitrile, flow rate: 400 nl/min) on a waters separating nanocolumn (nanoAcquity UPLC BEH130 C18 column, 0.075 mm ID*250 mm L, 1.7 m particle size, 130 Å pore size; column temperature: 40 °C). Peptides eluting from the column were analyzed in 2-s cycles selecting the most abundant multiply charged ions (*z* = 2–6, m/z range: 380–1580) for HCD fragmentation (normalized collision energy: 35%) following each MS1 scan. Both MS and MS/MS spectra were collected in the Orbitrap analyzer with a resolution of 120,000 or 15,000, respectively. Raw data were converted into peaklists using the Proteome Discoverer (v2.4 SP1) software. Proteins were identified using the BatchTag Web software of Protein Prospector (v6.3.1.) with the following parameters: database: Uniprot *Rattus norvegicus* sequences (2020.10.07. version, 36,457 sequences) concatenated with a randomized version for each entry and also supplemented with 172 additional sequences representing the most common contaminant proteins (such as trypsin and human keratins); enzyme: trypsin allowing maximum one missed cleavage site; modifications: static: methylthio on Cys, variable: cleavage of Met and/or acetylation of protein N-termini, oxidation of Met, pyroglutamic acid formation from peptide N-terminal Gln, deamidation of Gln or Asn, allowing up to two variable modifications/peptide; mass accuracy: 5 and 20 ppm for precursor and fragment ions, respectively defined as monoisotopic values. Acceptance parameters: minimum score: 22 and 15, maximum *E*-value: 0.01 and 0.05 on the protein and peptide level, respectively; minimum protein best discriminant score: 0. Peptide level false discovery rate was below 1% for all samples as estimated by the incidence of randomized sequence identifications.

Quantification of the proteins across the sample groups was performed using spectral counting. Three sham-operated control and nine BCCAO-operated rats were compared. Peptide counts of proteins were normalized to the total peptide count found in each sample. Proteins were removed from analysis with more than 1 or 6 missing values among the control or operated rats, respectively. Protein abundance mean ratios of BCCAO and control samples were calculated. An independent two-tailed Student’s *t*-test was performed on proteins of more than 1.5 or less than 0.5 mean ratios. The Benjamini–Hochberg procedure was applied with a false discovery rate of 0.25. In Online resource 1, we show the original *P*-values of only those proteins that remained significant after the procedure.

### Hierarchical Clustering and Principal Component Analysis

Hierarchical clustering and principal component analysis (PCA) were based on significantly altered proteins. PCA was performed in STASTICA 8.0 (StatSoft Inc., Tulsa, OK, USA); the calculations of gel-based proteomics and MS-based proteomics data were based on the correlations of normalized fluorescence intensities and normalized protein abundances, respectively. For the clustering of gel-based proteomics and MS-based proteomics data, z-scores of normalized fluorescence intensities and normalized protein abundances were calculated, respectively. Then, hierarchical clustering was performed, and heatmaps were created with R v4.0.5 programming language in RStudio v2022.07.1 with the heatmap.2 function of gplots package which uses agglomerative hierarchical clustering.

### Enrichment Analyses of Altered Proteins

Enrichment analysis was performed on altered proteins of MAM, mitochondria, and CSF, using the DAVID Bioinformatics Resources v2022q1 [26, 27] with the background of the rat genome. The functional annotation tool of DAVID was used to evaluate the enrichment of the KEGG pathway, GO biological process, cellular component, and molecular function terms with the EASE score and count threshold of 0.01 and 2, respectively. Benjamini–Hochberg correction was applied for controlling the false discovery rate.

### Validations of Protein Changes by Western Blot

We carried out western blot experiments on mitochondrial (*n* = 8) samples to confirm our proteomic results. Samples (30 µg) were diluted with sample loading buffer (1 M Tris–HCl pH = 6.8, 8 w/v% SDS, 24 v/v% glycerol, 200 mM DTT, 0.2 v/v% bromophenol blue) and incubated at 96 °C for 5 min. Protein samples were separated on 10% acrylamide gel by SDS-PAGE. Then, the gel was transferred to a PVDF membrane. The membrane was washed with distilled water and stained with Ponceau S stain (0.5 (w/v)% in 1 (v/v)% acetic acid), after washing with distilled water; it was photographed when it air dried. The membrane was destained in 200 µM NaOH and 20% acetonitrile and washed again in distilled water, then blocked with 3% BSA solution (in TBS-T for 1 h at RT). The membrane was stained with primary antibodies: anti-Hibadh (at 1:1000 dilution) (Sigma-Aldrich, Cat# HPA021002), anti-P4hb (at 1:1000 dilution) (Sigma-Aldrich, Cat# HPA018884), anti-Hspa5 (at 1:1000 dilution) (Thermo Fisher Scientific, Cat# PA1-014A), and anti-Trap1 (at 1:2000 dilution) (Thermo Fisher Scientific, Cat# MA1-010) at 4 °C overnight and washed in TBS-T. Secondary antibody staining with A647-conjugated anti-rabbit (Jackson ImmunoResearch, Cat# 711–605-152) and A594-conjugated anti-mouse (Jackson ImmunoResearch, Cat# 715–585-151) was applied in 1:800 dilution for 2 h at RT and washed in TBS-T then TBS. The antibody-labeled membrane was scanned with Typhoon Trio + scanner (Amersham) with appropriate filter settings and 50-micron resolution. Images were analyzed with ImageJ (version 1.53c); densitometric values of protein bands were determined and normalized to the highest intensity band on the blot and then to the total protein content. A two-sample test for variance in OriginPro 9 was performed to test that the variances of the groups do not differ significantly (*P* < 0.05). Independent two-tailed Student’s *t*-test (*P* < 0.05) was applied to confirm altered levels of proteins in OriginPro 9.

## Results

### Magnetic Resonance Angiography (MRA) Confirmed the Efficacy of the Occlusions

**Fig. 1:**
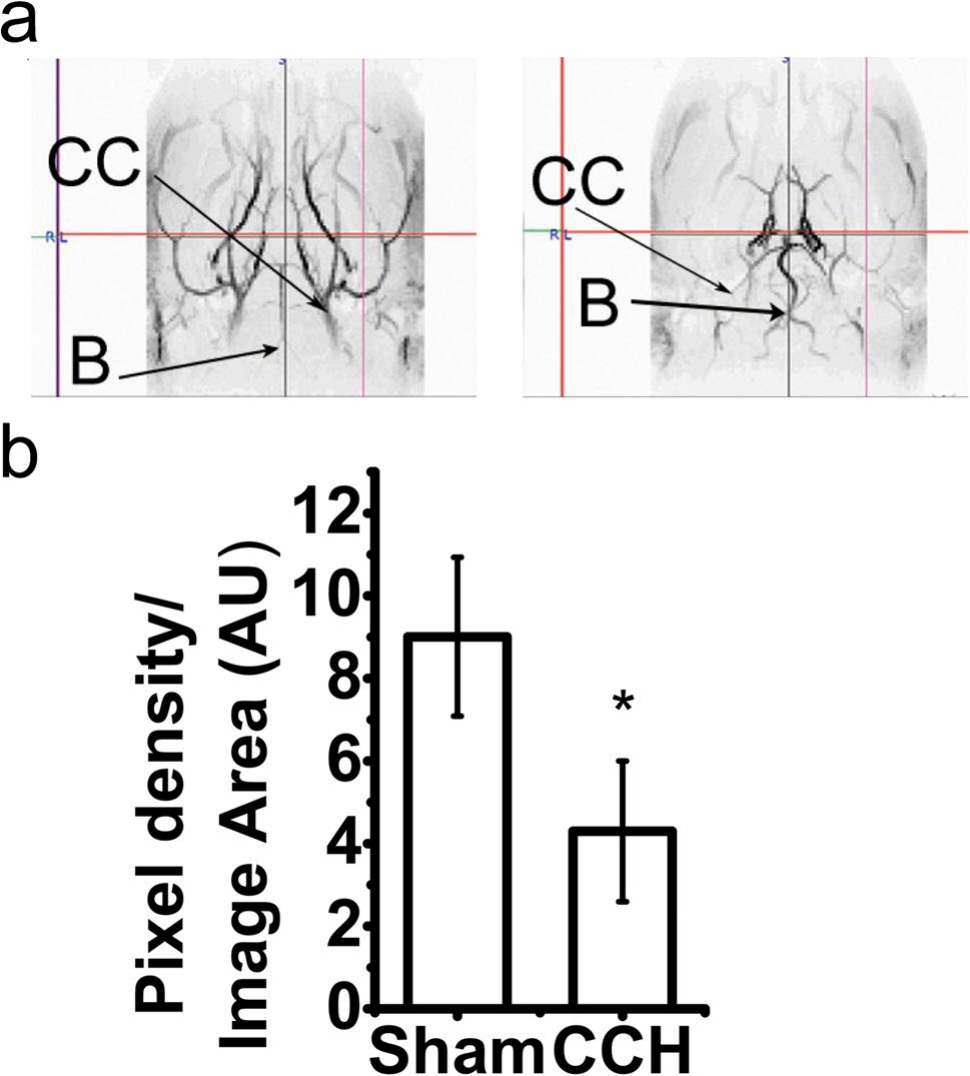
MRA recordings of animals 5 weeks after the operations. Representative images of sham-operated (left) and BCCAO (right) rats are shown (**a**). The common carotid arteries (CC) are occluded and the basilar artery (B) is thickened in the BCCAO animals. Image analysis of MRA recordings (**b**), statistical evaluation was performed with non-parametric, Mann–Whitney test (*: *P*-value < 0.01)

MRA confirmed that the experiment did not affect sham-operated animals, while the blood flow was bilaterally blocked in the common carotid artery in each CCH animal. As a result of the occlusions, the basilar artery thickened in the operated rats (Fig. 1a), implying it partially overtook the functions of the occluded arteries. Based on image analysis, the mean pixel density of blood vessels significantly (*P*-value = 0.0026) decreased in CCH animals 5 weeks after the surgery (Fig. 1b).

### Mitochondrial and MAM Proteome Alterations Induced by CCH

**Fig. 2:**
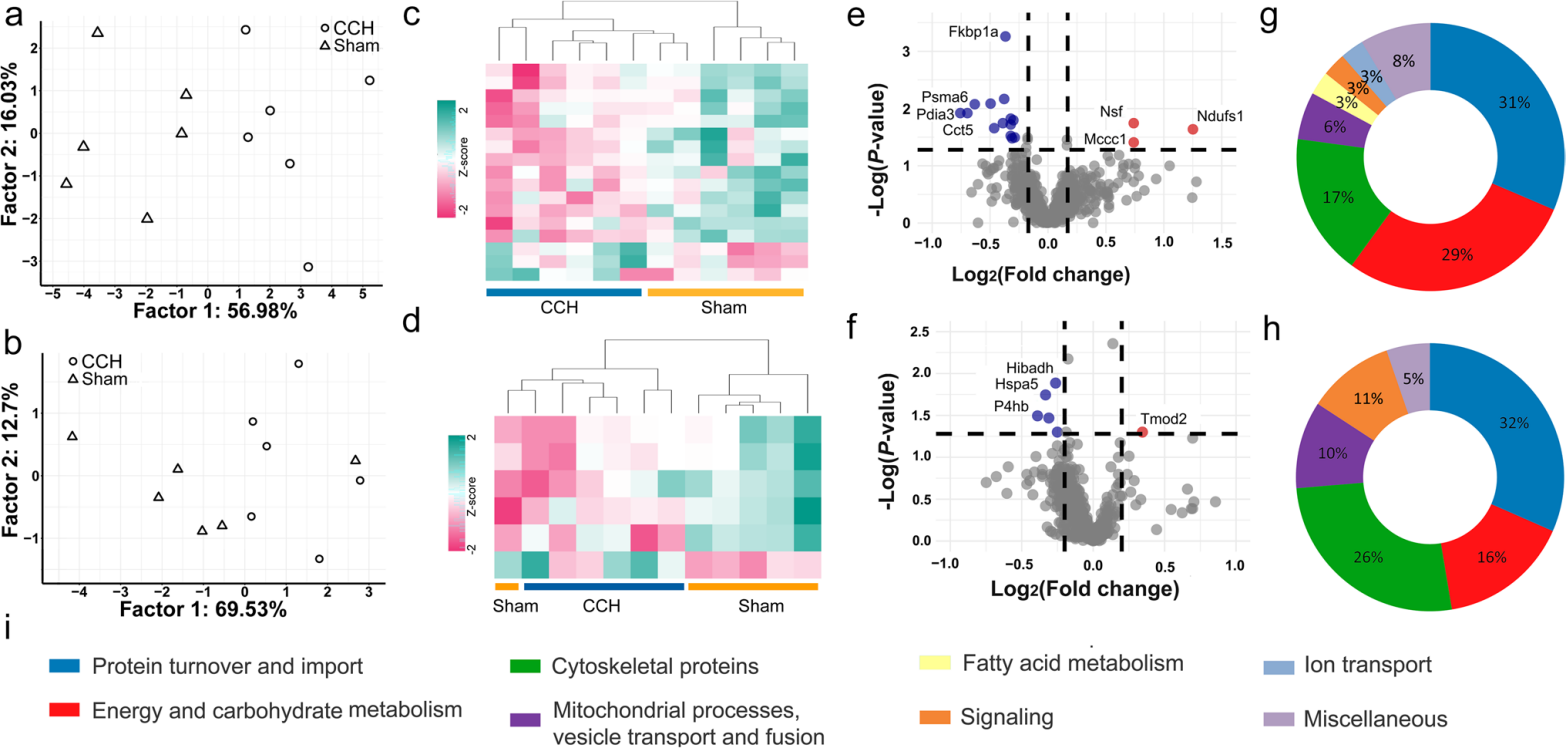
PCA (**a**, **b**) and hierarchical clustering (**c**, **d**) of MAM and mitochondria, respectively, are shown. Volcano plots of MAM (**e**) and mitochondria (**f**) proteomic results. Significantly decreased and elevated spots are shown in blue and red, respectively. Pie charts of significantly altered proteins in MAM (**g**) and mitochondria (**h**) represent functional annotation with a legend (**i**)

The current gel-based proteomics study was performed on the MAM and mitochondria containing subcellular fractions of frontal cortices from stepwise BCCAO rats. We have separated 725 and 708 protein spots on the gels of MAM and mitochondria samples, respectively. PCA and hierarchical clustering showed that CCH and sham-operated animals could be distinguished based on significantly altered proteins in the MAM (Fig. 2a and b). However, one sham-operated animal prominently differed from the rest in the mitochondrial samples (Fig. 2c and d). Seventeen and 6 spots showed significant change in the MAM and mitochondria sample of the CCH rats compared to the sham-operated group, respectively (Fig. 2e and f; Online Resources 1 and 2). In the MAM samples, altered proteins showed fold changes between − 1.69 to 2.38. Three spots showed elevation while 14 decreased compared to the control group. Strikingly, most of the altered proteins in the mitochondria decreased with fold changes (FC) varying between − 1.31 and − 1.2, and one spot increased with the fold change of 1.27. From altered spots, HPLC–MS/MS identified 19 proteins from the mitochondria sample and 35 from MAM (Table 1). It is known that several proteins can be present in one spot and one protein can be found in several spots, thus the number of significantly altered spots is not equal to the number of identified proteins. Taken together, we observed a modest alteration in the levels of mitochondrial proteins and a more complex proteome modification in the MAM-containing sample.

**Table 1:** List of altered proteins in subcellular fractions of MAM and mitochondria. Uniprot accession numbers, gene names, primary function, subcellular localization, and fold changes (FC) of significantly altered proteins in the MAM and mitochondria are shown

Accession No	Gene	FC	Protein name	Primary function	Subcellular localization
MAM					
Energy and carbohydrate metabolism					
Q66HF1	Ndufs1	2.38	NADH-ubiquinone oxidoreductase 75 kDa subunit, mitochondrial	Electron transport chain	Mitochondria
P10719	Atp5f1b	− 1.24	ATP synthase subunit beta, mitochondrial	ATP synthesis	Mitochondria
Q4FZZ4	Pdha1	− 1.41	Pyruvate dehydrogenase E1 component subunit alpha	Pyruvate metabolism	Mitochondria
A0A0G2JZH8	Pdhx	− 1.25	Dihydrolipoamide acetyltransferase component of pyruvate dehydrogenase complex	Pyruvate metabolism	Mitochondria
P07323	Eno2	− 1.24	Gamma-enolase	Glycolysis	Cytoplasm
P09117	Aldoc	− 1.31	Fructose-bisphosphate aldolase C	Glycolysis	Cytoplasm
P04797	Gapdh	− 1.38	Glyceraldehyde-3-phosphate dehydrogenase	Glycolysis	Cytoplasm
P48500	Tpi1	− 1.22	Triosephosphate isomerase	Glycolysis	Cytoplasm
D3ZVD3	Nt5c1a	− 1.41	5'-nucleotidase, cytosolic IA	Nucleotide metabolism	Cytoplasm
Protein turnover and import					
P63018	Hspa8	2.38	Heat shock cognate 71 kDa protein	Protein folding	Cytoplasm
F1M953	Hspa9	2.38	Stress-70 protein, mitochondrial	Protein folding	Mitochondria
P11598	Pdia3	1.69	Protein disulfide-isomerase A3	Protein folding	ER, Mitochondria
P63039	Hspd1	− 1.69	60 kDa heat shock protein, mitochondrial	Protein folding	Mitochondria
Q68FQ0	Cct5	− 1.62	T-complex protein 1 subunit epsilon	Protein complex assembly	Cytoplasm
Q5XIM9	Cct2	− 1.25	T-complex protein 1 subunit beta	Protein complex assembly	Cytoplasm
Q6P7B0	Wars	− 1.25	Tryptophan–tRNA ligase, cytoplasmic	Protein synthesis	Cytoplasm
Q6PDW1	Rps12	− 1.25	40S ribosomal protein S12	Protein synthesis	ER, Cytoplasm
P60901	Psma6	− 1.55	Proteasome subunit alpha type-6	Protein degradation	Cytoplasm
F1LP30	Mccc1	1.67	Methylcrotonoyl-CoA carboxylase subunit alpha, mitochondrial	Leucine catabolism	Mitochondria
P09606	Glul	− 1.31	Glutamine synthetase	Glutamine metabolism	Mitochondria, ER
Cytoskeletal proteins					
Q6P9V9	Tuba1b	− 1.69	Tubulin alpha-1B chain	Cytoskeletal protein	Cytoskeleton
Q5XIF6	Tuba4a	− 1.62	Tubulin alpha-4A chain	Cytoskeletal protein	Cytoskeleton
P68370	Tuba1a	− 1.62	Tubulin alpha-1A chain	Cytoskeletal protein	Cytoskeleton
P47942	Dpysl2	− 1.25	Dihydropyrimidinase-related protein 2	Cytoskeletal protein	Cytoskeleton
Q62952	Dpysl3	− 1.25	Dihydropyrimidinase-related protein 3	Cytoskeletal protein	Cytoskeleton
A0A0G2JUL7	Septin11	− 1.25	Septin 6 (Predicted), isoform CRA_b	Cytoskeletal	Cytoskeleton
Mitochondrial process and vesicle transport, fusion					
Q9QUL6	Nsf	1.67	Vesicle-fusing ATPase	Vesicle transport	ER, Golgi apparatus
D3ZJX5	Timm50	− 1.38	Mitochondrial import inner membrane translocase subunit TIM50	Mitochondrial import	Mitochondria
Fatty acid metabolism					
P55053	Fabp5	− 1.25	Fatty acid-binding protein 5	Fatty acid carrier	Extracellular region
Signaling proteins					
Q62658	Fkbp1a	− 1.29	Peptidyl-prolyl cis–trans isomerase FKBP1A	TGF beta signaling	Cytoplasm, Plasma membrane, ER
		− 1.23			
Ion transport					
P62815	Atp6v1b2	− 1.62	V-type proton ATPase subunit B, brain isoform	Proton transport	Lysosome, Endomembrane system
Miscellaneous					
Q9WU34	Septin3	− 1.41	Neuronal-specific septin-3	Unknown	Cytoskeleton
B5DFE0	Mpp6	− 1.62	Membrane palmitoylated protein 6	Unknown	Plasma membrane
Q5FVL2	Emc8	− 1.3	ER membrane protein complex subunit 8	Unknown	ER
Mitochondria					
Energy and carbohydrate metabolism					
P10719	Atp5f1b	− 1.2	ATP synthase subunit beta, mitochondrial	ATP synthesis	Mitochondrial inner membrane
M0R7U1	Ak5	− 1.2	Adenylate kinase 5	ATP metabolism	Cytoplasm
A0A0G2K401	Pcca	− 1.24	Propionyl-CoA carboxylase alpha chain, mitochondrial	Carbon metabolism	Mitochondrial matrix
Protein turnover and import					
Q5XHZ0	Trap1	− 1.24	Heat shock protein 75 kDa, mitochondrial	Protein folding	Mitochondrial inner membrane
P06761	Hspa5	− 1.26	Endoplasmic reticulum chaperone BiP	Protein folding	Mitochondria, ER
P04785	P4hb	− 1.31	Protein disulfide-isomerase	Protein folding	Endoplasmic reticulum
Q5I0G4	Gars	− 1.24	Glycine–tRNA ligase (Fragment)	Protein synthesis	Mitochondrial matrix
P24268	Ctsd	1.27	Cathepsin D	Endopeptidase	Lysosome
P29266	Hibadh	− 1.29	3-hydroxyisobutyrate dehydrogenase, mitochondrial	Valine catabolism	Mitochondrial matrix
Cytoskeletal proteins					
P47942	Dpysl2	− 1.24	Dihydropyrimidinase-related protein 2	Cytoskeleton regulation	Cytoskeleton
P85108	Tubb2a	− 1.31	Tubulin beta-2A chain	Cytoskeletal protein	Cytoskeleton
B4F7C2	Tubb4a	− 1.31	Tubulin beta chain	Cytoskeletal protein	Cytoskeleton
Q6P9V9	Tuba1b	− 1.31	Tubulin alpha-1B chain	Cytoskeletal protein	Cytoskeleton
P70566	Tmod2	1.27	Tropomodulin-2	Cytoskeleton regulation	Cytoskeleton
Mitochondrial process and vesicle transport, fusion					
F1LX07	Slc25a12	− 1.24	Solute carrier family 25 member 12	Aspartate-glutamate transport	Mitochondrial inner membrane
P61765	Stxbp1	− 1.2	Syntaxin-binding protein 1	Synaptic vesicle fusion	Mitochondria, Plasma membrane
Signaling proteins					
P12369	Prkar2b	− 1.31	cAMP-dependent protein kinase type II-beta regulatory subunit	PKA signaling	Cytoplasm
Q5XI34	Ppp2r1a	− 1.2	Protein phosphatase 2 (Formerly 2A), regulatory subunit A (PR 65), alpha isoform, isoform CRA_a	Signaling	Plasma membrane, mitochondria
Miscellaneous					
O88954	Mpp3	− 1.24	MAGUK p55 subfamily member 3	Unknown	Plasma membrane

Since the MAM comprises the interacting surfaces of the endoplasmic reticulum (ER) and mitochondrial membrane, the subcellular fraction of MAM will inevitably contain some mitochondrial proteins. Here, the spot with the highest fold change (*FC* = 2.38) consisted of NADH dehydrogenase (Ubiquinone) Fe-S protein 1 (Ndufs1) (the subunit of complex I of the electron transport chain), and heat shock proteins, namely stress-70 protein, mitochondrial (Hspa9 or mortalin), and heat shock cognate 71 kDa protein (Hspa8). While, in the most decreasing spot (*FC* =  − 1.69), we detected protein disulfide isomerase A3 (Pdia3) chaperone among others with lower peptide count. The other two increased spots, with fold changes of 1.67, are comprised of methylcrotonoyl-CoA carboxylase subunit alpha (Mccc1) and vesicle-fusing ATPase (Nsf) which are involved in branch-chained amino acid catabolism and vesicle transport, respectively. All the other protein spots decreased; T-complex protein 1 subunit epsilon (Cct5), proteasome subunit alpha type-6 (Psma6), and neuronal-specific septin-3 (Septin3) had fold changes of − 1.62, − 1.55, and − 1.41, respectively. In the mitochondrial fraction, the spot decreasing and increasing the most (*FC* =  − 1.31 and 1.27) comprised protein disulfide isomerase (P4hb or Pdia1) chaperon and cytoskeletal protein tropomodulin-2 (Tmod2), respectively. Other altered spots changed between − 1.20 and − 1.29; these contained endoplasmic reticulum chaperone BiP (Hspa5), ATP synthase subunit beta (Atp5f1b), heat shock protein 75 kDa (Trap1), and cytoskeletal protein dihydropyrimidinase-related protein 2 (Dpysl2).

The annotation of significantly altered proteins can reveal the disturbed cellular processes in the MAM and mitochondria induced by the stepwise BCCAO (Fig. 2g–i). Protein turnover and import showed the highest ratio of altered proteins in the MAM (*n* = 11; 32%) and mitochondria samples (*n* = 6; 32%). Energy and carbohydrate metabolism-associated proteins were highly represented in the MAM (*n* = 10; 29%) and less robustly in the mitochondria (*n* = 3; 16%) of the frontal cortex. Additionally, we identified cytoskeletal proteins in both mitochondria (*n* = 5; 26%) and MAM (*n* = 6; 18%) subcellular fractions. Proteins of fatty acid metabolism (*n* = 1; 3%) and ion transport (*n* = 1; 3%) were present only in MAM. Proteins involved in membrane dynamics and mitochondrial processes were also present among the altered proteins of MAM (*n* = 2; 6%) and mitochondria (*n* = 2; 10%). In conclusion, our data show a wide disturbance of the protein turnover process and mitochondrial energy metabolism in response to stepwise BCCAO.

### Functional Proteomic Alterations in the CSF Reflect Cellular Organelle Changes in the Frontal Cortex

**Fig. 3:**
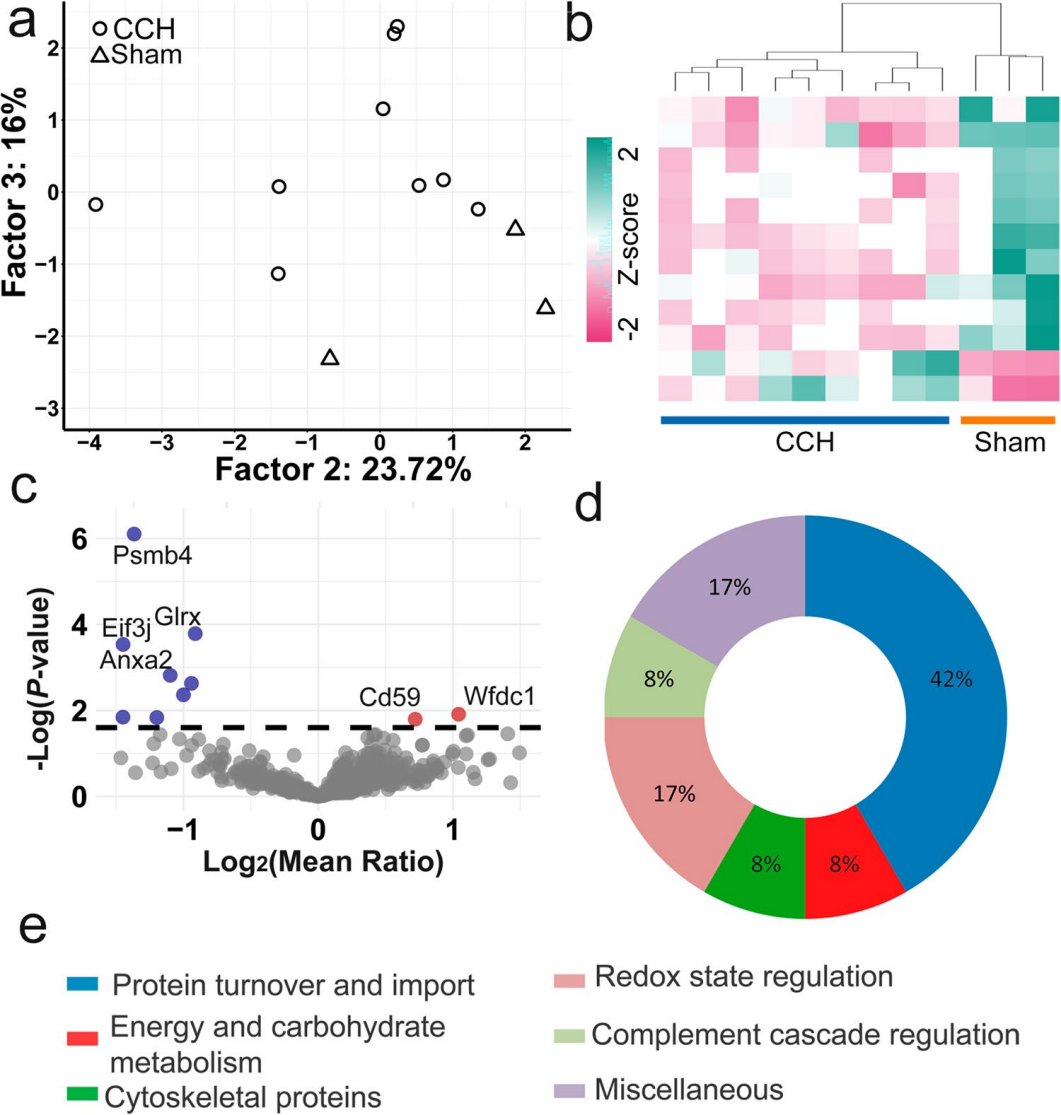
PCA (**a**) and hierarchical clustering (**b**) of CSF could separate sham-operated and CCH animals. Volcano plots of CSF proteomic results (**c**) and pie chart (**d**) of functional annotation with a legend (**e**) are shown. Significantly decreased and elevated spots are shown in blue and red in the volcano plot, respectively

PCA and hierarchical clustering could distinguish CCH and sham-operated animals based on significantly altered proteins in the CSF (Fig. 3a and b). We found 12 proteins with significantly altered abundance with mean ratios between 0.32 and 2.06, using MS-based proteomics (Fig. 3c, Table 2, and Online Resource 1). Two of them, CD59 glycoprotein (Cd59) and WAP four-disulfide core domain protein 1 (Wfdc1), increased by 1.65 and 2.06 fold, respectively. Annexin A2 (Anxa2) showed the lowest mean ratio of 0.32. Functional annotations (Fig. 3b) showed that 17% of altered proteins were involved in redox state regulation, namely glutaredoxin-1 (Glrx) and glutathione S-transferase P (Gstp1) decreased by 0.53 and 0.52. Protein turnover and import had the highest ratio of altered proteins in the CSF (*n* = 5; 42%) similar to the MAM and mitochondria. Four of them were less than halved compared to control namely, proteasome subunit beta (Psmb4), ubiquitin carboxyl-terminal hydrolase isozyme L3 (Uchl3), eukaryotic translation initiation factor 3 subunit J (Eif3j), and importin subunit beta-1 (Kpnb1)); and Wfdc1, an endopeptidase inhibitor, increased.

**Table 2:** List of altered proteins in the CSF and their primary cellular functions and localization. Uniprot accession numbers, gene names, primary function, subcellular localization, and mean ratios of significantly altered proteins in the CSF are shown

Accession No	Gene	Mean ratio	Protein name	Primary function	Subcellular localization
CSF					
Energy and carbohydrate metabolism					
P47967	Lgals5	0.467	Galectin-5	Carbohydrate binding	Cytoplasm
Protein turnover and import					
G3V8U9	Psmb4	0.388	Proteasome subunit beta	Protein degradation	Cytoplasm
Q91Y78	Uchl3	0.366	Ubiquitin carboxyl-terminal hydrolase isozyme L3	Protein degradation	Cytoplasm
A0JPM9	Eif3j	0.342	Eukaryotic translation initiation factor 3 subunit J	Protein translation	Cytoplasm
P52296	Kpnb1	0.436	Importin subunit beta-1	Nuclear protein import	Nucleus envelope
O70280	Wfdc1	2.059	WAP four-disulfide core domain protein 1	Endopeptidase inhibitor	Extracellular space
Cytoskeletal proteins					
Q08163	Cap1	0.5	Adenylyl cyclase-associated protein 1	Cytoskeleton regulation	Cytoskeleton
Redox state regulation					
P04906	Gstp1	0.521	Glutathione S-transferase P	Detoxification of Reactive Oxygen Species	Mitochondria, Cytoplasm
Q9ESH6	Glrx	0.531	Glutaredoxin-1	Redox state regulation	Cytoplasm, Mitochondria
Complement cascade regulation					
P27274	Cd59	1.647	CD59 glycoprotein	Inhibitor of the complement membrane attack complex	Plasma membrane
Miscellaneous					
Q499R7	Ppa1	0.366	Inorganic diphosphatase (Fragment)	Hydrolysis of pyrophosphate	Cytoplasm
Q07936	Anxa2	0.321	Annexin A2	Membrane raft assembly	Plasma membrane

### Enrichment Analysis of Altered Proteins Upon Stepwise BCCAO Is in Accordance with Functional Annotation

**Fig. 4:**
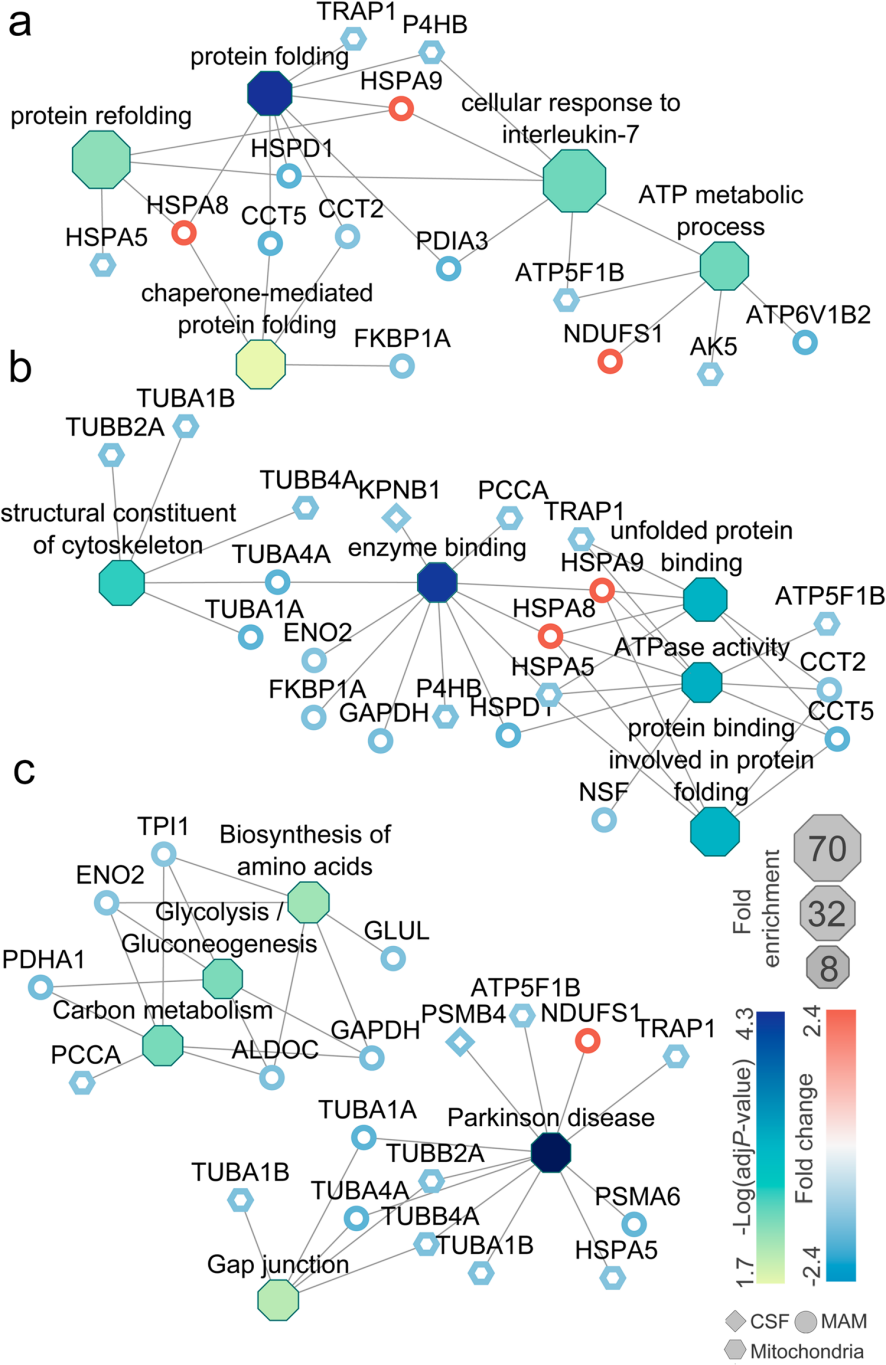
Enrichment map of top five terms of GO biological process (**a**), GO molecular function (**b**), and KEGG pathway (**c**) is connected to their related proteins labeled with gene names. –Log(adjusted *P*-value) of terms and fold changes of related proteins are color coded. Fold enrichments of terms are represented with node size. Altered proteins of MAM, mitochondria, and CSF are depicted with circle, hexagon, and rhombus, respectively

The enrichment of gene ontology (GO) terms and Kyoto Encyclopedia of Genes and Genomes (KEGG) pathways can give further insight into the disturbed cellular process and related genes affected by stepwise BCCAO (Online Resource 3). Enrichment maps of the top five terms of GO biological process, molecular function, and KEGG pathways are shown in Fig. 4. Regarding biological process (Fig. 4a), ATP metabolic process was enriched with five related genes Hspa8, Atp5f1b, v-type proton ATPase subunit B (Atp6v1b2), Ndufs1, adenylate kinase 5 (Ak5) (32.2 fold, adj. *P*-value = 4.2E-03). Also, protein folding was significantly overrepresented (20.8 fold, adj. *P*-value = 5.44E-05). In accordance, protein binding involved in protein folding molecular function term was highly enriched (32.2 fold, adj. *P*-value = 9.02E-04) with five related genes (Hspa9, Hspa8, Cct2, Hspa5, Cct5) (Fig. 4b). Among KEGG pathways (Fig. 4c), the most enriched terms were glycolysis/gluconeogenesis (14.3 fold, adj. *P*-value = 4.83E-03) and biosynthesis of amino acids (12.5 fold, adj. *P*-value = 7.25E-03). Also, pathways of neurodegeneration in multiple diseases were enriched (4.5 fold, adj. *P*-value = 2.24E-03) with 11 related genes (such as Trap1, Psma6, Psmb4, Atp5f1b, Hspa5, Ndufs1a, and tubulins) (Online Resource 3).

### Altered Proteins of Organellar Quality Control Validated by Western Blot

**Fig. 5:**
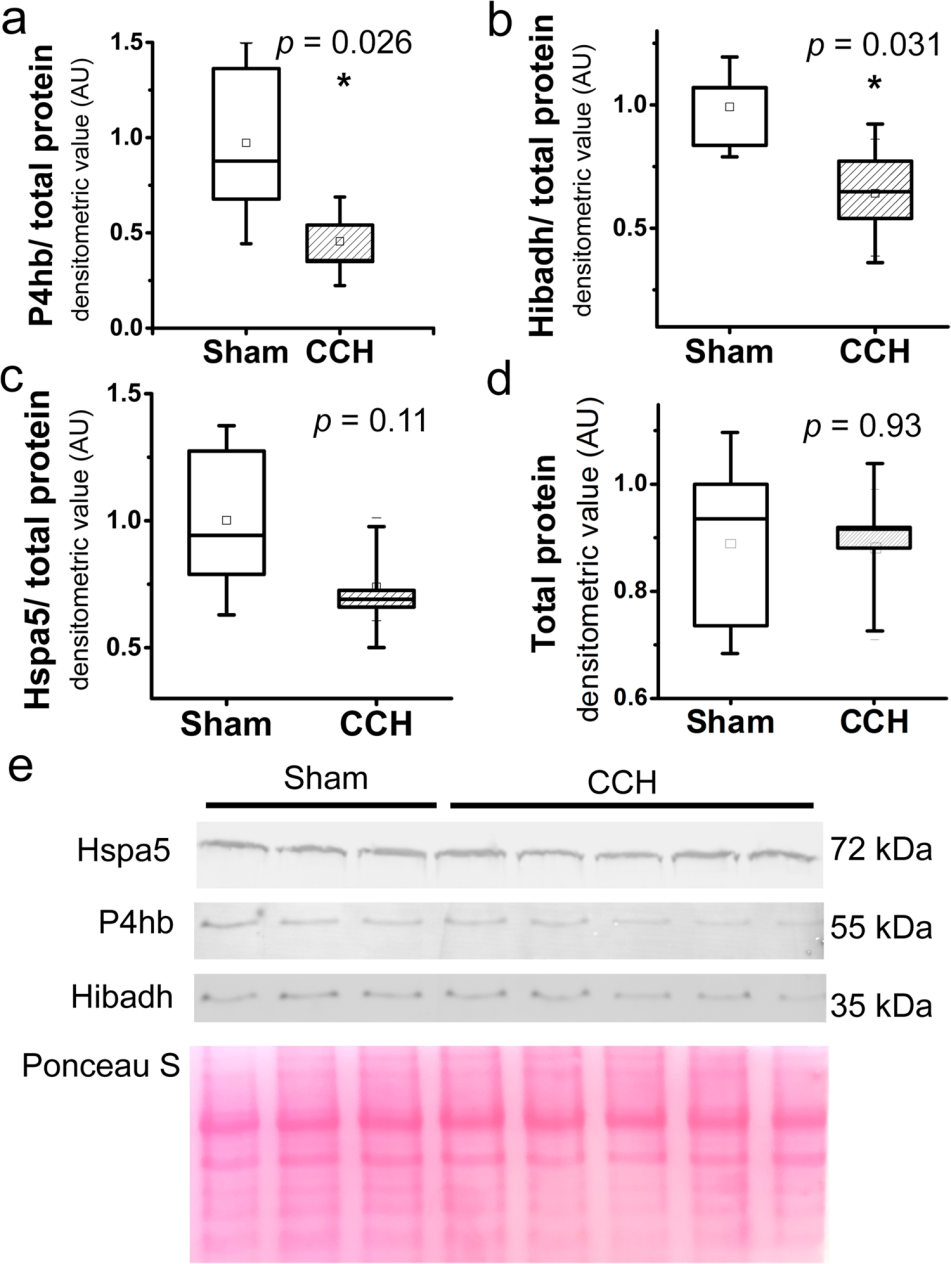
Altered levels of P4hb (**a**), Hibadh (**b**), and Hspa5 (**c**) in the mitochondria analyzed by western blot. Specific protein bands and total protein staining (**d**, **e**) are shown. Independent two-tailed Student’s *t*-test was applied (*: *P*-value < 0.05)

We analyzed the levels of four proteins involved in mitochondrial and ER quality control and protein folding by western blot to confirm our proteomic results (Fig. 5a–e). We performed a two-sample test for a variance to ensure equal variances of the two experimental groups, and we did not find significant changes in variances (for Hibadh, *P* = 0.74; for P4hb, *P* = 0.16; for Hspa5, *P* = 0.41; and for total protein, *P* = 0.57). P4hb (*P*-value = 0.026) and Hibadh (*P*-value = 0.031) levels decreased significantly in the mitochondria (Fig. 5a–b). While the decline of the Hspa5 level in the mitochondria was not significant (*P*-value = 0.11) (Fig. 5c). We did not find significant changes in total protein content in the blot (*P*-value = 0.93) (Fig. 5d).

## Discussion

### Limitations

High-throughput methods are powerful tools to track molecular changes upon different treatments in animals, tissues, or cell lines. Two-D DIGE proteomics enables the separation of two or three protein samples labeled by different fluorescent dyes simultaneously on a single gel. This technique makes spot comparison and protein quantification more reliable and reproducible [28].

At the same time, 2-D DIGE has some limitations that must be taken into account. Hydrophobic membrane proteins solubilize poorly in polar detergent-free solvents applied in isoelectric focusing (IEF). This effect aggravates their migration into the gel; thus, their detection is limited. Additionally, we could not separate and detect the higher molecular weight proteins and the strongly acidic or alkaline ones since applied IEF and PAGE allows protein separation in the range of ~ 3 to ~ 10 isoelectric points (pI) and 10–100 kDa, respectively. In addition, highly abundant proteins can mask lower-abundant protein alterations if more than one protein runs into the same spot. This can be improved by subcellular fractionation, such as mitochondria and MAM isolation in this study. However, contaminations from other cellular organelles are inevitable and not negligible [29]. At the same time, the applied preparations are highly reproducible and validated [25].

### Chaperons of the ER Protein Quality Control

MAM is a specialized area of the ER, communicating directly with mitochondria. ER is essential in protein synthesis, folding, and trafficking, and several ER chaperones localize to the MAM [30]. Upon acute energy restriction, misfolded and unfolded proteins accumulate in the ER lumen [31]. In response to ER stress, the unfolded protein response (UPR) is triggered to restore proteostasis by reducing protein synthesis, promoting proper protein folding, and the degradation of misfolded proteins. ER-associated degradation (ERAD) induces misfolded protein clearance by proteasomal degradation. Thus, ERAD and UPR serve to restore normal proteostasis upon stress.

A widely used marker of ER stress is the Hspa5 chaperone; we detected its decrease in the mitochondria. Although the Hspa5 level showed a declining trend analyzed by western blot, its decrease was not significant. Overexpressing Hspa5 in primary cultured astrocytes protected mitochondria against ischemic stress [32]. Additional ER chaperones implicated in UPR, Pdia3, and P4hb, also changed in our study, and the latter was validated by western blot. Pdia3 reduced ER stress upon ischemia [33], and its mRNA level was upregulated upon glucose depletion [34]. P4hb is upregulated in short-term hypoxic astrocytes and protects neurons from apoptotic cell death in vitro [35]. Chaperone-containing T-complex (TRiC) protein subunits are chaperones that compose the CCT-β complex upon interacting with β-tubulin. Disruption of the CCT-β complex reduced ER stress response through altering mitochondrial membrane potential and inducing heat shock protein 75 kDa (Trap1) dependent protein degradation and ERAD [36]. In our study, we detected decreased levels of T-complex protein subunits 2, 5 (Cct2, Cct5), several β-tubulin subunits (Tubb2a, Tubb4a), and Trap1. It was found that a component of ERAD localizes proteasome subunits to the ER [37], and we detected decreased proteasome subunit alpha type-6 (Psma6) levels in the MAM and proteasome subunit beta (Psmb4) in CSF.

In a CCH model, EPTA staining of hippocampal slices revealed abnormal protein aggregation of newly synthesized proteins in the cytosol, 3 months after the operation [38]. In addition, elevated levels of ubiquitinated proteins were shown in the chronic BCCAO model [39], implying that disturbed proteostasis is a long-term effect of CCH. In conclusion, most of the proteins of the quality control mechanism decreased in our study, implying that ER stress response is hindered in response to chronic hypoperfusion, and its protective role in maintaining normal proteostasis might be diminished.

We detected dihydropyrimidinase-related proteins 2 and 3 (Dpysl2 and 3) which were also identified in the MAM fraction previously [29, 40]. However, their role in the regulation of MAM is poorly studied.

### Altered Proteins of Mitochondrial Oxidative Phosphorylation and Quality Control

The occlusion of the common carotid arteries reduces cerebral blood flow for several weeks in BCCAO rats. Although the blood flow recovers to the control level [3], subsequently, mitochondrial damage develops [41]. Additionally, BCCAO augmented the level of reactive oxygen species and reduced the activity of Mn-superoxide dismutase (Sod2), even 8 weeks after the surgery [5]. Two components of the oxidative phosphorylation (OXPHOS) system were altered in our study; one of them is the ATP synthase subunit beta (Atp5f1b), and the other is Ndufs1. Altered levels of subunits of the electron transport chain can hinder the correct assembly of the OXPHOS system which can lead to oxidative [42] and possibly proteotoxic stress [43].

Mitochondrial quality control is crucial in maintaining normal cellular metabolism. Several proteins and molecular pathways serve to maintain or restore mitochondrial functions such as chaperones, proteases, and import proteins involved in mitochondrial UPR (mtUPR). In the mitochondria, misfolded or damaged proteins are digested, and the peptides are transported to the cytosol where they induce mtUPR [44]. In our proteomic study, we found 60 kDa heat shock protein (Hspd1 also known as Hsp60) and Hspa9 that are involved in mtUPR. Hspa9 is a matrix chaperone and the only component of the mitochondrial import complex that has an ATPase function [45]. Since most of the mitochondrial proteins are encoded in the nucleus, the import and refolding of proteins are essential to maintain mitochondrial proteostasis. Loss of Hspa9 induced proteotoxic stress and autophagic clearance of damaged mitochondria in vitro [46].

Trap1 is a mitochondrial chaperone, involved in oxidative stress response. It reduces mitochondrial respiration, promotes glycolysis, and reduces reactive oxygen species [47]. We have detected decreased level of Trap1 in mitochondria in response to long-term hypoperfusion by proteomics analyses.

Cathepsin D (Ctsd) is a lysosomal endopeptidase and its release from lysosomes triggers bax activation [48], mitochondrial cytochrome C release, and transmembrane potential loss during apoptosis upon oxidative stress [49]. We detected Ctsd elevation in the mitochondrial fraction.

Surprisingly, several cytoskeletal proteins were detected in the mitochondrial samples. However, α- and β-tubulin were shown to associate with mitochondrial membranes [50]. Furthermore, tubulins decreased mitochondrial respiration by reducing the permeability of voltage-dependent anion channel for ADP [51, 52]. Reducing mitochondrial respiration protects against oxidative stress; in our study, levels of several tubulin subunits dropped in mitochondria and MAM samples. This might indicate increasing oxidative damage and increased level of ROS found in the BCCAO model [5].

### Metabolism of Branch-Chained Amino Acids (BCAAs) in Dementia

BCAAs (Leu, Ile, Val) are essential amino acids necessary for protein synthesis and also have roles in the regulation of metabolic functions. BCAAs serve as nitrogen donors for amino acids (e.g., glutamine) and neurotransmitter synthesis (e.g., glutamate, gamma-aminobutyric acid).

Decreased levels of BCAAs were detected in the serum levels of patients with Alzheimer’s disease and dementia [53]. Furthermore, decreased level of leucine (Leu) was also detected in the saliva of Alzheimer’s disease and vascular dementia patients [54]. We have detected an increased level of methylcrotonoyl-CoA carboxylase subunit alpha (Mccc1) which is responsible for the catabolism of Leu. However, we also measured a decreased level of 3-hydroxyisobutyrate dehydrogenase (Hibadh), an enzyme of valine catabolism, and its decrease was validated by western blot. Thus, our results suggest unbalanced BCAA catabolism in CCH.

### Disturbed Protein Turnover and Redox State Regulation are Reflected in the CSF

While we could not detect significant changes of the same proteins in the CSF and the subcellular fractions, several proteins of protein turnover decreased in the CSF and subcellular fraction proteomes upon stepwise BCCAO, such as Eif3j, Uchl3, Psmb4 in the CSF, Psma6 in the MAM, and glycine–tRNA ligase (Gars) in the mitochondria.

Glrx and Gstp1 have main roles in the detoxification of reactive oxygen species which levels’ are elevated upon BCCAO [5]. Although we did not detect significant alterations of Glrx and Gstp1 in the subcellular fraction of the frontal cortex, both proteins decreased in the CSF in our study, and they were shown to localize to mitochondria [55, 56]. Furthermore, we previously showed altered Gstp1 level in the synaptosomal fraction of the occipital cortex in CCH rats compared to sham-operated controls [23].

There were several proteins that we could only detect in sham-operated animals (see Online Resource 1). For instance, proteasome subunit beta type-7, exportin-1, and electron transfer flavoprotein subunit beta have roles in protein degradation, protein transport from the nucleus, and mitochondrial electron transport, respectively. Lack of their detection can indicate that their levels are below the detection limit or missing from the samples of BCCAO animals. Thus, it further suggests that protein turnover impairment and mitochondrial damage of the brain can be detected in the CSF.

## Conclusions

Here, we studied the long-term effects of oxidative stress in the brain by inducing CCH and investigating the proteomic alterations in two subcellular fractions of the frontal cortex and the CSF. Our results suggest declining cellular response against oxidative and proteotoxic stress since several key proteins of these processes showed decreasing levels, such as Glrx, Gstp1, T-complex protein subunits, Hspd1, and P4hb. Stress responses have a protective effect on cells; therefore, we could speculate that the long-term decline of stress responses can be one of the causes of cognitive impairment during chronic cerebral hypoperfusion. We can also suggest impaired protein turnover by detecting reduced levels of a proteasome subunit and a eukaryotic translation initiation factor in the CSF and amino acid-tRNA ligases in the MAM and mitochondria. Reduced protein turnover might be a compensatory mechanism to reduce energy consumption and oxidative damage in response to impaired blood flow. In conclusion, we have detected reduced levels of several components of protein turnover in the mitochondria, MAM, and CSF of the stepwise BCCAO model, implying that altered cellular processes of brain tissue can be detected in the cerebrospinal fluid by proteomic analysis.

## Supplementary Information

Online resource 1. The list of significantly altered proteins in the CSF and identified proteins of spots in the mitochondria and MAM (XLSX 80 kb)

Online resource 2. Representative gel images of the proteomics analysis with significantly altered protein spots in the (a) MAM and (b) mitochondria (red and blue spots indicate increasing and decreasing results, respectively) (PNG 3036 kb)

High Resolution Image (TIF 34917 kb)

Online resource 3. Result of enrichment analysis using DAVID Bioinformatics Resources v2022q1 (EASE score threshold: 0.01, Count threshold: 4, Background: rat genome) (XLSX 20 kb)

## Data Availability

The datasets supporting the conclusions of this article are available upon request.

## Abbreviations

*CCH*: Chronic cerebral hypoperfusion
*CBF*: Cerebral blood flow
*VD*: Vascular dementia
*AD*: Alzheimer’s disease
*BCCAO*: Bilateral common carotid artery occlusion
*MAM*: Mitochondrial-associated membrane
*2-D DIGE*: 2-Dimensional differential gel electrophoresis
*HPLC–MS/MS*: High performance liquid chromatography-tandem mass spectroscopy
*MRA*: Magnetic resonance angiography
*IB-1*: Isolation buffer-1
*IB-2*: Isolation buffer-2
*IB-3*: Isolation buffer-3
*MRB*: Mitochondria resuspending buffer
*IEF*: Isoelectric focusing
*FC*: Fold change
