# Supplementary figures and images for: Chronic Cerebral Hypoperfusion-Induced Disturbed Proteostasis of Mitochondria and MAM Is Reflected in the CSF of Rats by Proteomic Analysis
Source: Mol Neurobiol. 2023 Feb 21;60(6):3158–74. doi: 10.1007/s12035-023-03215-z (PMC10122630; doi:10.1007/s12035-023-03215-z)

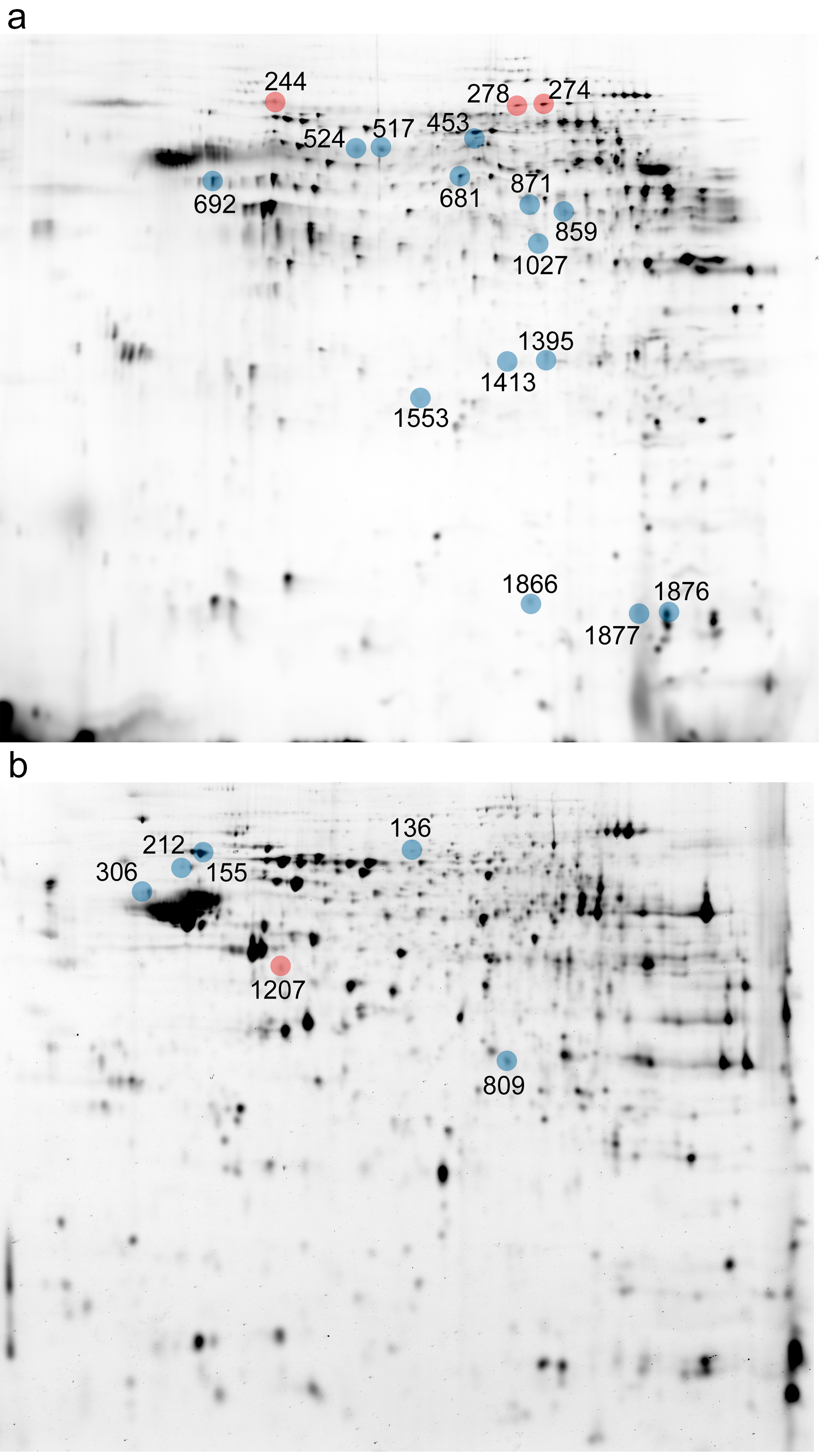

Supplement: Supplementary file 2 — Representative gel images of the proteomics analysis with significantly altered protein spots in the (a) MAM and (b) mitochondria (red and blue spots indicate increasing and decreasing results, respectively) (PNG 3036 kb) [file 12035_2023_3215_Fig6_ESM.png]

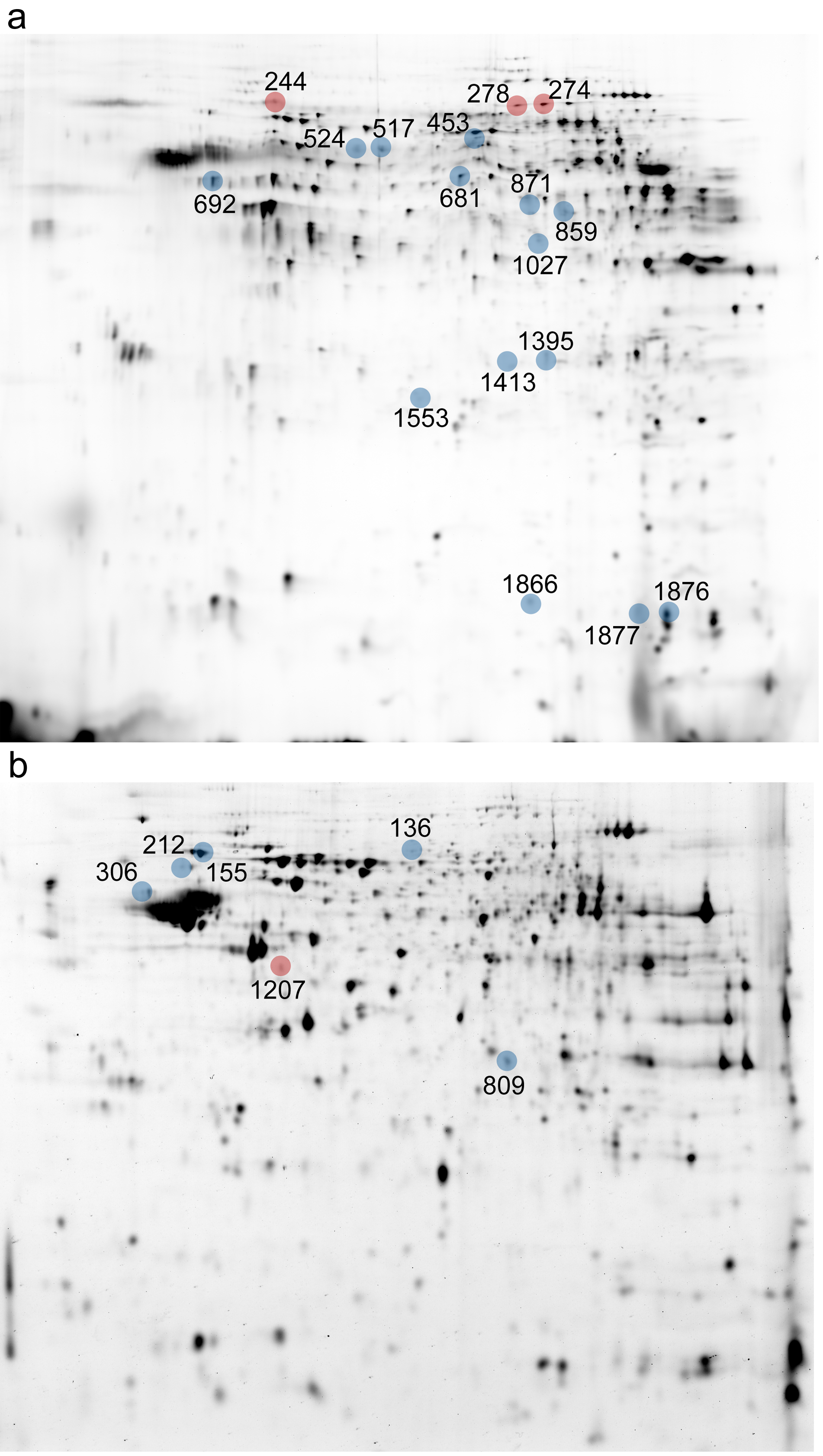

Supplement: Supplementary file 3 — High Resolution Image (TIF 34917 kb) [file 12035_2023_3215_MOESM3_ESM.tif]
